# Critical commentary on the association between thiazolidinedione use and dementia risk in patients with type 2 diabetes

**DOI:** 10.1111/1753-0407.13409

**Published:** 2023-05-08

**Authors:** Uzayr Wasif, Usmaan Al‐Shehab, David F. Lo

**Affiliations:** ^1^ Department of Biology Rutgers, the State University of New Jersey New Brunswick New Jersey USA; ^2^ Department of Medicine Rowan University School of Osteopathic Medicine Stratford New Jersey USA; ^3^ American Preventive Screening & Education Association (APSEA) Stratford New Jersey USA

We read with pleasure the article by Zhao et al^1^ titled “Thiazolidinedione use is associated with reduced risk of dementia in patients with type 2 diabetes mellitus: A retrospective cohort study” and would like to provide critical commentary on the inclusion of modifiable and nonmodifiable risk factors for dementia in subgroup analyses for patients taking thiazolidinediones (TZDs). We hope that these insights may guide further research and improvement in the association between TZDs and dementia risk.

Zhao et al investigated the association between TZD use and the risk of dementia. The article presents a retrospective population‐based cohort study conducted in mainland China to evaluate the association between TZD use and the risk of dementia in a Chinese population with T2DM. The study used an active‐comparator new‐user design to evaluate the association between TZD use and the risk of dementia in the Chinese population with T2DM. The study included new users of TZDs and alpha‐glucosidase inhibitors (AGIs), commonly used oral glucose‐lowering agents at the same stage of T2DM in China. New use of TZDs or AGIs was defined as the first prescription of a drug from either class with no fill of TZDs and AGIs in a baseline washout period of 6 months. The date of the first prescription was defined as the index date. Participants aged <18 years old were excluded from the cohort, as were participants who received combination treatment of TZDs and AGIs at the index date and who had received a diagnosis of any dementia before the index date. Patients who had no consecutive prescriptions of these drugs within 6 months of the index date were also excluded.

First, it is important to consider that recent studies have highlighted the association between age of diabetes onset and increased risk of dementia.^2^ To better understand this relationship, it is essential to account for potential confounding variables such as age at diagnosis. Therefore, we propose conducting a subgroup analysis of study participants based on their age at diabetes diagnosis to determine whether this variable influences the risk of dementia. By doing so, we can assess the extent to which age of diabetes onset may be an independent risk factor for dementia and potentially inform strategies for early intervention and prevention.

Second, it is important to consider the effect that mental health may have on the onset of dementia. A recent study conducted in New Zealand provides evidence that mental health is a risk factor for dementia, and the study further concludes that the association between mental disorders and dementia was larger than the association between physical diseases and dementia.^3^ Additional research is required, where a subgroup analysis would be implemented to compare the onset of dementia in patients with a history of mental illness and patients without a history of mental illness. Overall, this subgroup analysis would help determine whether or not mental illness may be a confounding variable in the onset of dementia.

Lastly, the study relies on administrative databases for identifying incident dementia cases and prescription drug use. These databases may have limitations in terms of accuracy and completeness, which could affect the validity of the results.^4^ Additionally, the study does not take into account the participants’ full medication history, the only stipulation being that the participant must be a new user of TZD; this may produce unknown confounding factors. We must also consider that the study is retrospective, which limits the control over the data collection process and the quality of the available data. This may lead to erroneous conclusions and calls into question the validity of the results.^5^

Zhao et al shed new light on the relationship between TZDs and onset of dementia. The study's novel approach of analyzing the role of TZDs in dementia risk provides valuable insights for future research in this field. We look forward to seeing this future publication and hope to see some of the confounding variables listed herein amended. We encourage larger scale studies that can examine the long‐term effects of TZDs on dementia risk across different populations, and explore potential interactions with other factors such as lifestyle and genetics.

## FUNDING INFORMATION

No funding was received for this study/paper.

## CONFLICT OF INTEREST STATEMENT

The authors declare that they have no competing interests.
